# First detection of one of the tick-borne lymphadenopathy (TIBOLA) etiological agent in ticks from a highly frequented sub-urban forest near Paris, France

**DOI:** 10.1371/journal.pgph.0005705

**Published:** 2026-01-12

**Authors:** Eva A. Krupa, Laurine Levillayer, Matthieu Prot, Artem Baidaliuk, Richard E. L. Paul, Etienne Simon-Loriere, Sarah I. Bonnet

**Affiliations:** 1 Institut Pasteur, Université Paris Cité, CNRS UMR 2000, INRAE USC 1510, Ecology and Emergence of Arthropod-borne pathogens unit, Paris, France; 2 Institut Pasteur, Université Paris Cité, CNRS UMR 2000, Evolutionary Genomics of RNA Viruses unit, Paris, France; 3 Institut Pasteur, Université Paris Cité, Bioinformatics and Biostatistics Hub, Paris, France; Qatar University College of Medicine, QATAR

## Abstract

In the context of global changes including climate, environmental and socio-economic modifications, the surveillance of tick populations in term of species distribution and harboured pathogens is an absolute necessity. With this aim in view, ticks were collected in May 2022 in an highly frequented suburban forest located near Paris, France. The objective was to identify tick species and tick-borne pathogens that may warrant long-term monitoring, as well as to assess added value of metatranscriptomic Next Generation Sequencing (mNGS) for the detection of known and possibly new pathogens in ticks. Both *Dermacentor reticulatus* and *Ixodes ricinus* were collected. In addition to expected pathogens detected in *I. ricinus* (i.e., *Anaplasma, Babesia,* and *Borrelia* species), we report the detection of *Rickettsia conorii subsp. raoultii*, a zoonotic bacterium never identified in the region before and transmitted by a tick species on the rise: *D. reticulatus*.

## Author summary

Surveillance of tick populations is increasingly important in the present context of global changes to detect unexpected transmitted pathogens in new areas. In this preliminary survey, the collection of a small number of ticks (n = 40) allowed detection of classically identified pathogens in the forests surrounding Paris, France, but also for the first time a bacterium responsible for Tick-borne lymphadenopathy (TIBOLA).

## Introduction

Ticks are known vectors of multiple pathogens affecting both veterinary and human health worldwide [1]. In Europe and France, *Ixodes ricinus*, a multi-host hard tick, vector of various tick-borne pathogens (TBP), is widely distributed and commonly found in deciduous and mixed-forest [2]. *Dermacentor reticulatus,* another common European tick species*,* has been recognized as recently expanding geographically thanks to its high adaptiveness to a wide range of habitats and climatic conditions [3]. Despite being less studied, this species also poses an important threat because of its ability to transmit TBP, including the spotted-fever-group *Rickettsia* species, *Babesia canis* as well as Omsk haemorrhagic fever and tick-borne encephalitis viruses [3]. *Dermacentor reticulatus* preferentially feeds on small rodents during the larval and nymph stages and on larger mammals as adults. Recorded hosts include wild and domesticated carnivores, sheep, cattle, and horses. This species can be found in a wide range of habitats, including those shared with *I. ricinus,* but it favors open areas [3]. Currently, the geographical distribution of several tick species including *I. ricinus* and *D. reticulatus* -and their associated pathogens- is changing due to socio-economic and environmental changes including climatic factors, as well as the intensification of human and animal movements [4]. *Ixodes ricinus* is the most abundant tick species, occurring in a large geographic range in Europe, with several studies documenting its northward and altitudinal expansion, likely linked to climate change, but changes in hosts and landscape appear also important [4–7]. The distribution of *D. reticulatus* is considered mosaic or highly focal in Europe and in France, although the species appears to be present throughout the territory. However, there is a lack of data in many regions [8,9].

In France, most studies on ticks have focused on *I. ricinus* and related TBPs [2]. Some of these studies have been conducted at regular intervals in a peri-urban forest located near Paris, the Sénart forest (48° 40’ N, 2° 29’ E), due to its high frequentation level (almost 3 millions visitors per year) and thus presenting a high risk environment for the human population [10–16]. This large peri-urban forest of more than 3000ha is predominantly composed of deciduous trees such as oaks (*Quercus* sp*.*), chesnut trees (*Castanea sp*.), birch (*Betula* sp.), beech (*Fagus sylvatica*) and hornbeam (*Carpinus* sp.) and harbours a variety of vertebrate hosts for ticks (rodents, wild boar (*Sus scrofa*), roe deer (*Capreolus capreolus*), introduced tamias (*Tamias sibiricus*)). This area of France is classified by the Köppen climate classification as Temperate with no dry season and warm to hot summers. As mentioned, this forest has been regularly investigated regarding tick presence since 2005 and, while *I. ricinus* has always been the main collected tick species, the presence of *D. reticulatus*, *Ixodes acuminatus*, and *Ixodes trianguliceps* has been also recorded [10–16]. TBP have been detected using targeted PCR in *I. ricinus* collected both as questing ticks and on hosts revealing the presence of various pathogens such as *Anaplasma* sp*.*, including *Anaplasma phagocytophilum*; *Babesia venatorum (*formerly *B.* sp*. (EU1)*, *B. divergens*, *B. capreoli*; *Bartonella* sp. including *B. birtlesii*; *Borrelia burgdorferi* s.l, including *B. burgdorferi* sensu stricto, *B. garinii*, *B. afzelii*, *B. valaisiana*, *B. spielmanii*, *B. afzelii/valaisiana*, *B. garinii/lusitaniae*; *B. miyamotoi*; *Rickettsia helvetica*, *R. felis* and *Francisella tularensis* [10–12,15,16]. However, until now, no studies have been carried out on pathogens potentially carried by *D. reticulatus*, the second most frequent species in this forest.

The aim of the present preliminary study was to screen TBP present in both *I. ricinus* and *D. reticulatus* as part of a rapid cross-sectional survey, 5 years after the last published collection in the same area [11]. Our aim was to determine whether long-term monitoring is warranted for the future, and also allowed assessment of the added value of metatranscriptomic Next Generation Sequencing (mNGS) for the detection of unexpected and potentially new pathogens in both well studied (i.e., *I. ricinus*) and under-studied (i.e., *D. reticulatus*) tick species in France.

## Materials and methods

### Study area and tick collection

Access authorization for tick collection was given by official letter from the “Office National des Forêts”. Questing ticks (nymphs and adults) were collected in Sénart forest (48° 40’ N, 2° 29’ E) in May 2022 between 16:30 and 17:30 by 5 collectors using the flagging method. The collectors dragged 50 × 60 cm cotton cloths over the vegetation for 1 hour. The weather was sunny with 24 °C. Ticks were brought back to the laboratory alive. Ticks were identified morphologically using taxonomic keys [17] and by MALDI-TOF by using 2 tick legs according to the protocol described in Boyer *et al.* [18], and then stored at -80 °C until further analysis.

### RNA extraction

Ticks were crushed individually for both nymphs and adults in 150 µL of lysis buffer from NucleoSpin RNA Plus kit (Macherey-Nagel, Switzerland), with six metal beads and a pinch of glass powder, using a Precellys Evolution homogeniser (Bertin technologies, France) at 6500 rpm for 2 × 20 s with 5 s pause, repeated twice. RNA was then extracted using the NucleoSpin RNA Plus kit following the manufacturer’s protocol. RNA samples were eluted in 60 µL of RNAse-free water and stored at -80°C until use. RNA were converted to cDNA using M-mlv reverse transcriptase (Invitrogen, USA). Both RNA and cDNA quantity and quality was assessed using NanoDrop One (Thermo-Fisher, United-States of America). In addition, RNA extraction and reverse transcription efficiencies were confirmed in all samples with polymerase chain reaction (PCR) amplification of the 16S rRNA mitochondrial gene using tick-specific primers 16S-f (5’-CCGGTCTGAACTCAGATCAAGT-3’) and 16S-r (5’-GCTCAATGATTTTTTAAATTGCTGT-3’) [19]. PCR conditions were modified and included an initial denaturation step at 95 °C for 5 min followed by 35 cycles for 30 s at 93 °C, 30 s for

primer annealing at 53 °C, and 30 s for primer extension at 72 °C. A final extension step was carried out for 7 min at 72 °C.

### Polymerase chain reaction amplification for TBP

Dedicated conventional PCR assays targeting the 16S gene of *Borrelia burgdorferi* s.l, 16S gene of *Anaplasma* spp., Citrate synthase (gltA) gene of Spotted fever group of *Rickettsia* spp., gltA gene of *Bartonella* spp., putative prolactin-inducible protein (ppIp) gene of *Francisella tularensis* and the 18S gene of *Babesia* spp*.* were performed as previously described [12]. Negative (sterile water) and positive controls corresponding to targeted DNA cloned in plasmids were included in each run. Sanger sequencing (Eurofin genomic, Cologne, Germany) was performed for positive samples after PCR product purification from a 2% agarose gel using a PCR clean-up Gel extraction kit (Macherey-Nagel, Oensingen, Switzerland) according to the manufacturer’s protocol. Sequences obtained were compared with known sequences listed in the GenBank nucleotide sequence databases via the National Center for Biotechnology Information BLAST search option.

### Next generation sequencing

RNA samples were DNase-treated (Qiagen, Courtaboeuf, France), prior to cDNA synthesis using random hexamers (Thermo Fisher Scientific, Asnières-sur-Seine, France) and the superscript IV (Thermo Fisher Scientific). Second strand synthesis was performed using the NEBNext Second Strand Synthesis buffer, DNA polymerase I, DNA ligase and RNase H (NEB France, Évry-Courcouronnes). Libraries were prepared using the Nextera XT kit (Illumina, Paris, France) according to the manufacturer’s protocol, and sequenced on a NextSeq 500, using a mid-output 150 cycles kit (paired-end). Sequencing reads were quality trimmed using trimmomatic v0.39 [20] and *de novo* assembled with metaSPAdes v3.15.2 [21]. Assembled contigs were queried against the NCBI nr database using Diamond (v 2.1.9). Sequences were analysed in search of tick-borne pathogens. In addition to hits for *Borrelia*, *Babesia* and *Anaplasma* spp., one *D. reticulatus* sample presented hits to *Rickettsia* spp. The closest BLASTn hits in NCBI nt database (99.25% nt identity) to one of the identified contigs was *Rickettsia conorii* subsp*. raoultii.* Due to the unexpected identification of this bacterium, we performed additional analysis of the sequence of R. *conorii* subsp*. raoultii*. We then extracted the *de novo* assembled contigs that corresponded to 16S and 23S rRNA gene sequences of *R. conorii* subsp*. raoultii* strain Khabarovsk (NZ_CP010969) using Geneious v2023.2.1 (https://www.geneious.com) mapping tool and confirmed the coverage by read mapping with bowtie2 v2.3.5.1 [22].

### Phylogenetic analysis

The two contigs were subjected to megablast search to the nt NCBI database (on Oct 16, 2025), and top hits (>97% nt identity, > 1000 nt length) were extracted for phylogenetic analysis. Additionally, we extracted 16S and 23S genes from the newly deposited *R. conorii* subsp. *raoultii* genome assemblies (PRJNA1136696), trimming chimeric assembly artifacts. Multiple alignments were prepared with mafft v1.5.0 (G-INS-i) and maximum-likelihood trees were inferred using IQ-TREE v2.0.6 [23] with ModelFinder [24] and UFBoot2 (1000 bootstrap replicates) [25], and visualized with FigTree v1.4.4 (http://tree.bio.ed.ac.uk/software/figtree/). Alignments are provided as supplementary material. Trees inferred from 16S and 23S sequences are available in S1 and S2 Fig respectively.

## Results

Forty questing ticks were collected, resulting in an average number of 8 ticks per collector per hour, of which 34 were identified as *I. ricinus* and six as *D. reticulatus.* Morphological identification and MALDI-TOF identification matched on 100% of ticks tested (**Table 1**). Following RNA extraction, 1.01 to 34.1 ng/µL of RNA were obtained per tick. After the RT, cDNA were diluted to a concentration of 6.75 + /-1.15ng/µL before use. Four ticks were found positive for at least one pathogen and confirmed through conventional PCR amplification and mNGS (**Table 1**). Specifically, one male *I. ricinus* was found positive for *Anaplasma phagocytophilum*. Two *I. ricinus* nymphs were positive for *Babesia* spp.: one for *B. divergens* and the other for *B. venatorum*. This second nymph was also co-infected with *Borrelia afzelii*. One female *D. reticulatus* was positive for a spotted-fever-group *Rickettsia* sp., identified as *R. conorii* subsp*. raoultii*.

**Table 1:** Number of ticks collected per species and life-stages and associated detected pathogens.

Tick species	Life-stage	Number of collected ticks	Tick-borne pathogen (TBP) detected	Accession number
*Ixodes ricinus*	Female	3	0/3	
	Male	4	*Anaplasma phagocytophilum* (1/4)	OQ622281(16S)
	Nymph	27	*Babesia divergens* (1/27) *Borrelia afzelii/Babesia venatorum* co-infection (1/27)	OQ622167 (18S) OQ622282 (16S)/ OQ622166 (18S)
*Dermacentor reticulatus*	Female	4	*Rickettsia conorii* subsp*. raoultii* (1/4)	OQ813759 (gltA) PP442177 (16S rRNA) PP442186 (23S rRNA)
	Male	2	0/2	
**Total**		**40**		

The obtained pathogen sequences were submitted to Genbank and the corresponding accession numbers mentioned in the Table.

**Fig 1:**
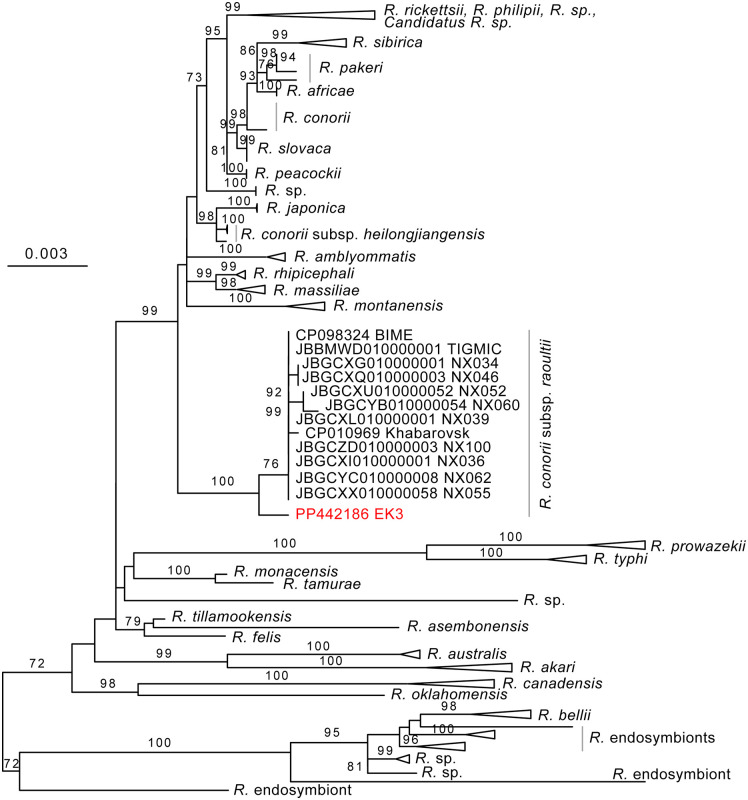
Phylogenetic tree of 23S rRNA sequence of *Rickettsia* species, including the *R. conorii* subsp. *raoultii* collected in Sénart forest (highlighted in red). Branch support values above 70 are indicated above each branch. The tree is represented with midpoint rooting. The clades with high support values are collapsed where possible and the species or subspecies are indicated next to the clades. In the *R. conorii* subsp. *raoultii* clade, identical sequences are omitted for visualization purposes. The non-collapsed tree is available in S2 Fig.

The 16S rRNA sequence obtained by mNGS was identical to multiple sequences of isolates of *R. conorii* subsp*. raoultii* (e.g., Kaliningrad-1 – Kaliningrad-31, Bashkortostan-1, Crimea-1, Stavropol, Tomsk, BL029-2 isolates). However, *R. conorii* subsp. *raoultii* was not monophyletic on the 16S gene tree. Therefore, we focused on 23S for a more robust analysis. Our novel sequence formed a monophyletic clade with *R. conorii* subsp*. raoultii* strain (**Fig 1**), in a basal position, and showed 99.7-99.9% identity to the 23S of *R. conorii* subsp*. raoultii* strain BIME (CP098324), strain Khabarovsk (CP010969) and sequences with available 23S annotations from PRJNA1136696 (see S1 and S2 Text).

The rRNA sequences were submitted to GenBank (see **Table 1**. for accession numbers) and the raw reads to the Sequence Read Archive (SRA) under project number PRJNA1077891.

## Discussion

This preliminary study, whose purpose was not to determine tick densities or real prevalences of pathogens therein but to validate the interest of monitoring a frequently visited suburban forest, allowed to observe 4 infected ticks upon 40 collected over a short time. The collection was carried out at a season, time, and temperature at which the species of ticks likely to be present would be active [26]. Despite their preference for open areas, which are, within Sénart forest, less common, some adult *D. reticulatus* were collected during the survey and could then be analysed; the larval and nymphal stages being endophilic cannot be collected by flagging [8].

Previous studies performed on *I. ricinus* in Sénart forest reported *Borrelia* spp*.* and *A. phagocytophilum* as the most prevalent pathogens (prevalence ranging from 3.7% to 32% and from 1.5% to 10.8%, respectively), while *Babesia* spp*.* were more rarely detected (prevalence from 1.5% to 2%) [10–12]. In the present report, too few ticks were collected and analysed to evaluate prevalence. However, the same previously detected TBP were identified in *I. ricinus*: *A. phagocytophilum* was detected in a male *I. ricinus*, *B. divergens* was detected in one nymph, and another nymph was co-infected with *Borrelia afzelii* and *Babesia venatorum*. Tick co-infection with multiple pathogens was found to occur frequently, which poses a serious challenge for diagnosis and appropriate treatment [27]. *Babesia* spp*.* parasites are more likely to be acquired by nymphs or adult ticks feeding on large mammals, but can also be transmitted from the female to eggs and larvae, and then detected in unfed nymphs as reported here and elsewhere [28]. Both *Babesia* species can be pathogenic for humans with symptoms being fever and haemolytic anaemia [29]While it is not surprising to detect *B. venatorum* in the forest due to the presence of roe deer, the primary reservoir of this parasite [30], the detection of *B. divergens* in an area without cattle supports the hypothesis that wild reservoirs for this parasite may exist [10]. Regarding *A. phagocytophilum*, few human cases are diagnosed in France and occur mainly in the eastern part of the country; however, it is believed that the prevalence of this pathogen is increasing nationwide [31]. The identification of B. *afzelii* is also not surprising*,* as it is, with *B. garinii,* the bacteria most frequently involved in human cases of Lyme borreliosis in Europe, and the most frequently detected species in the northeastern quarter of France [2]. Infection by *B. afzelii* can result as Lyme disease symptoms, usually as a cutaneous lesion called *Erythema migrans* but can also worsen into neuroborreliosis or acrodermatitis chronica atrophicans [32].

Regarding *Rickettsia* species, only *Rickettsia helvetica* and *R. felis* have been found in previous studies in *I. ricinus* in the Sénart forest, at an intermediate to low prevalence (2.3% to 5.8%) [10–12,16] and 0.1%, respectively [11] compared with other pathogens detected in this forest. In the present study, one of the six *D. reticulatus* collected tested positive for *R. conorii* subsp*. raoultii*, marking the first mention of this pathogen in this location. Rickettsiae are obligate intracellular alpha-proteobacteria distributed worldwide, and ticks are known vectors of rickettsiae species responsible for spotted fever syndrome in humans. This syndrome is caused by at least 15 different *Rickettsia* species (SFG) including *R. conorii* subsp*. raoultii* [33]. This bacterium, first described in 2008, is present in Europe and Asia and is one of the agent that can cause SENLAT (scalp eschars and neck lymphadenopathy after a tick bite), also known as Tick-borne lymphadenopathy (TIBOLA) or Dermacentor-borne-necrosis erythema-lymphadenopathy (DEBONEL). Symptoms include fever, headache, rash, neck lymphadenopathy and an erythematous skin lesion at the site location of the tick bite, when on the scalp [34,35]. It is considered by some authors as an emerging TBP and whilst easily treated with doxycycline or chloramphenicol, unusual symptoms have been recently reported in China [36]. Incidence of TIBOLA cases is reported to increase in the north-east of France [37].

Both PCR and mNGS methods gave similar results in this preliminary study, resulting in a list of six common pathogens detected. These pathogens were targeted by dedicated PCR as they were previously described in literature covering this region, highlighting the need to monitor areas over the long term. mNGS however allowed a direct identification to the species of the pathogen (while PCR was targeting genus or group of species and an additional step of sequencing was necessary). mNGS also enabled the detection of the microbiota of ticks (including *Midichloria mitochochondrii* or *Francisella opportunistica*) which could be of interest [38]. Additionally, phylogenetic analysis from mNGS results on 23S gene show that the detected strain of *R. raoultii* is close to the BIME strain from China and another strain from Russia, suggesting that *R. raoultii* is shared across northern Europe and Asia, albeit additional sequences from various regions across Europe and from other genes are required to confirm this hypothesis. The phylogenetic tree also shows that this strain is located far away from another causative agent of TIBOLA namely *R. slovaca*.

The transmission of *R. conorii* subsp*. raoultii* by *D. reticulatus* has been experimentally validated [39], but the bacterium is believed to be transmitted by several other species of the *Dermacentor* genus including *D. marginatus, D. nuttalli,* and *D. silvarum* as well as by other genera such as *Haemaphysalis, Rhipicephalus, Hyalomma*, and *Amblyomma* [40]. In Europe, prevalence of *R. conorii* subsp. *raoultii* in *D. reticulatus* range from 28% to 50% in England, Germany, and Poland [41–44]. In France, similar prevalence has been reported in *Dermacentor* spp. with 31% in 2010–2011 and 20% in 2022 in Belle-Île-en-Mer, an island off the Atlantic coast of Brittany in western France [45,46]. In the South of France, *R. raoultii* has been detected in 23% of *Dermacentor* ticks removed from people from 2002 to 2013 [47]. More recently, Boyer et al. have reported a prevalence of 20% of *R. raoultii* in *D. reticulatus* collected in several areas of Eastern France in 2018 and 2019 [48].

Although this is the first mention of *R. conorii* subsp*. raoultii* in the Ile-de-France region, because *Dermacentor* spp. have been hitherto excluded from surveys, it is unclear whether its introduction into this region is recent or not. This finding raises the possibility that bacteria other than those classically implicated may be involved in rickettsial diseases around Paris. It is important to mention that the use of mNGS enabled this detection, and also provided insights on the phylogenetic relationship of the strain with the currently known diversity of *R. conorii* subsp*. raoultii.* As this study is limited in both time and space, further analysis of ticks should include analysis of *Dermacentor* sp. ticks in order to validate the presence of *R. raoultii* by estimating its prevalence in ticks in the area.

In conclusion, given that *D. reticulatus* is currently expanding its geographical range [8,49], and because we detected here for the first time a causative agent of SENLAT, this study underscores the need for continuous monitoring of the highly frequented forests surrounding Paris to survey TBP circulation and infrequently collected tick species. In addition, with changing distribution of tick species, methods such as mNGS could be invaluable for comprehensive surveillance of TBP, including unexpected or unknown agents. Their presence can remain undetected even in well monitored studies areas, and the movement of hosts and ticks can lead to the introduction of novel or non-recorded TBP. In a rapidly changing world, understanding the extent of the circulation of both tick species and TBP, as well as their role in causing clinical diseases in animals and humans, is crucial for the implementation of future prevention and control measures.

## Supporting information

S1 Fig Non collapsed tree inferred from 16S sequences of Rickettsia species. (EPS)

S2 Fig Non collapsed tree inferred from 23S sequences of *Rickettsia* species. (EPS)

S1 Text Raw data of alignment of 16S sequences of *Rickettsia* species, including Genbank ID and corresponding sequences. (TXT)

S2 Text Raw data of alignment of 23S sequences of *Rickettsia* species, including Genbank ID and corresponding sequences. (TXT)
